# Adjuvant chemotherapy in pancreatic cancer at different AJCC stages: a propensity score matching analysis

**DOI:** 10.1186/s40001-023-01572-y

**Published:** 2023-12-19

**Authors:** Xiao-hui Li, En-liang Zhou, Xiao-yuan Dong, Chong-yu Zhao, Yuan-xia Han, Bo-kang Cui, Xiao-jun Lin

**Affiliations:** 1 Department of Pancreatobiliary Surgery, State Key Laboratory of Oncology in South China, Guangdong Provincial Clinical Research Center for Cancer, Sun Yat-sen University Cancer Center, 651 Dongfengdong Road, Guangzhou, 510060 China; 2 Department of Gynecology, Guangdong Hydropower Hospital, Guangzhou, 510060 China; 3 Department of Hepatobiliary Surgery, The Second Affiliated Hospital of Army Medical University, Chongqing, 400037 China

**Keywords:** Adjuvant chemotherapy, Pancreatic cancer, Survival analysis, American Joint Committee on cancer

## Abstract

**Objective:**

In the treatment of resectable pancreatic cancer, adjuvant chemotherapy is viewed as essential. However, it is yet unclear how well adjuvant chemotherapy works at different illness stages. This study aims to investigate the efficacy of adjuvant chemotherapy in various pancreatic cancer stages.

**Materials and methods:**

Patients with pancreatic cancer who underwent surgical intervention at Sun Yat-sen University Cancer Center between January 2018 and January 2021 were included in this retrospective analysis.

**Results:**

168 patients were divided into two groups: the group receiving adjuvant chemotherapy (AC) and the group receiving independent surgery (no-AC). Survival analysis reveals that among stage I patients, the AC group demonstrates significant superiority over the no-AC group in terms of recurrence-free survival (RFS) and overall survival (OS) (P = 0.0028; P = 0.022). While there was no discernible difference in RFS between the AC and no-AC groups for patients with stage II illness (P = 0.69), the AC group significantly outperformed the no-AC group in terms of OS (P = 0.047). There was no discernible difference in RFS or OS between the AC and no-AC groups for patients with stage III pancreatic cancer (P = 0.40 and P = 0.20, respectively).

**Conclusions:**

The administration of adjuvant chemotherapy has been shown to improve the prognosis of patients diagnosed with stage I and II pancreatic cancer. However, its efficacy is limited in individuals with stage III pancreatic cancer. Therefore, there is an urgent need to investigate and develop more effective therapeutic options for patients in the advanced stage.

## Introduction

Adjuvant chemotherapy ranks among the foremost measures to extend the survival of patients with pancreatic cancer following resection. Multiple large-scale randomized controlled trials have substantiated the advantageous impacts of adjuvant chemotherapy on the survival rate of pancreatic cancer patients [1–3]. However, the potential benefits of adjuvant chemotherapy for patients with varying stages of pancreatic cancer remain unclear. A multicenter cohort study [4] has demonstrated that adjuvant chemotherapy enhances long-term survival among patients with stage IB, IIA, IIB, and III pancreatic cancer. However, adjuvant chemotherapy does not confer a survival benefit to patients with stage IA pancreatic cancer. Furthermore, the presence of lymph node metastasis frequently correlates with a poorer prognosis in individuals with pancreatic cancer [5]. The potential benefits of adjuvant chemotherapy for patients with pancreatic cancer, regardless of the presence or absence of lymph node metastasis, remain unclear. In a recent study involving 612 patients with resectable pancreatic adenocarcinoma, it was demonstrated [6] that individuals with N2 lymph node metastasis did not exhibit a response to gemcitabine-based adjuvant chemotherapy. Many pancreatic cancer patients undergo surgical resection without receiving adjuvant chemotherapy, and the reasons behind this choice may involve multiple factors. One crucial factor is the unclear value of adjuvant chemotherapy across different stages of the disease. The primary objective of this study is to evaluate the survival benefits of adjuvant chemotherapy in pancreatic cancer patients across different stages. Patients were grouped based on whether they received adjuvant chemotherapy, and independent prognostic factors were analyzed. Subsequently, survival analyses were conducted on subgroups stratified according to different stages of pancreatic cancer, aiming to identify individuals who could potentially benefit from adjuvant chemotherapy. The findings of this study may inform clinical decision-making concerning the utilization of adjuvant chemotherapy in select pancreatic cancer patients.

## Data and methods

### Data sources and patient selection

Patients with pancreatic cancer who underwent surgery at Sun Yat-sen University Cancer Center from January 2018 to January 2021 were enrolled in this retrospective study. The inclusion criteria were as follows: (1) patients with pancreatic cancer who underwent surgery and whose diagnosis was confirmed via pathological examination; (2) patients with Eastern Cooperative Oncology Group (ECOG) scores of 0–1 before surgery; (3) patients who signed the informed consent form. The exclusion criteria were as follows: (1) only abdominal exploration was performed owing to distant metastasis; (2) death within 30 days after the operation; (3) preoperative neoadjuvant therapy; (4) combined with other malignant tumors; (5) incomplete clinical characteristics or follow-up data.

### Surgery and adjuvant chemotherapy

The surgical procedure was based on tumor location, tumor size, relationship of the tumor with surrounding important blood vessels, and regional enlarged lymph nodes, and the final decision was made by senior surgeons. R0 resection was defined as pathologically confirmed negative margins, R1 resection was defined as microscopic residual cancer, and R2 resection was defined as macroscopic residual cancer. After surgical resection, patients were evaluated for their physical condition, and adjuvant chemotherapy was initiated approximately 4 weeks after surgery. Three regimens were used for adjuvant chemotherapy: gemcitabine plus capecitabine (GP), mFOLFIRINOX, and S-1 (an oral 5-fluorouracil prodrug mixture of tegafur, gimeracil, and oteracil). In the GP regimen, gemcitabine at the dose of 1000 mg/m^2^ was intravenously infused over 30 min on days 1, 8, and 15 of each 28 day cycle for 6 cycles, and capecitabine at the dose of 1660 mg/(m^2^*d) was orally administered on days 1–21 of each 28 day cycle for 6 cycles. In the mFOLFIRINOX regimen, oxaliplatin at the dose of 85 mg/m^2^ was intravenously infused over 2 h, irinotecan at the dose of 150 mg/m^2^ was intravenously infused over more than 30–90 min on day 1, L-leucovorin at the dose of 400 mg/m^2^ was intravenously infused over 2 h on day 1, and 5-FU at the dose of 2400 mg/m^2^ on d1, continuous intravenous infusion for 46 h, repeated every 2 weeks, was administered until 24 weeks. In the S-1 regimen, S-1 at the dose of 80 mg/d was orally administered on days 1–28 and repeated every 6 weeks until 6 months. Clinical oncologists adjusted the drug dose according to the tolerance of patients.

### Follow-up and evaluation

Patients were reexamined after 1 month of surgery and subsequently every 3 months until 5 years after surgery if no recurrence was found. The reexamination encompassed blood routine tests, liver and kidney function assessments, pancreatic tumor marker evaluations, as well as upper abdominal computed tomography (CT) and magnetic resonance imaging (MRI) scans (plain scan + enhanced). The follow-up period extended until January 1, 2023, serving as the cutoff date. Adjuvant chemotherapy's efficacy was assessed based on overall survival (OS) and recurrence-free survival (RFS). RFS was defined as the time from surgery to the first recording of tumor recurrence or metastasis. OS was defined as the time from surgery to death from any cause or to the last follow-up. Major adverse events were evaluated according to the Common Terminology Criteria for Adverse Events (CTCAE) Version 5.0.

### Propensity score matching and statistical analysis

Kaplan–Meier survival curves were plotted using the R (version 4.1.2) and RStudio software. The IBM SPSS Statistics (version 25.0) software was used to compare the baseline data, and Cox proportional hazards regression models were used for univariate and multivariate analyses. Propensity score matching (PSM) was performed using the IBM SPSS Statistics (version 25.0) software. The specific PSM method is described as follows: Propensity score-matched analysis was performed using a multivariate logistic regression model based on sex, age, pathological diagnosis, tumor differentiation, tumor size, microvascular invasion, nerve invasion, preoperative Ca19-9 levels, preoperative CEA levels, preoperative TBIL levels, tumor location, AJCC stage 8, lymphatic metastasis, surgical type, revascularization, surgical time, amount of bleeding, perioperative blood transfusion, surgical margin, postoperative pancreatic fistula, and hospitalization time. Patients who received or did not receive adjuvant chemotherapy were grouped in a ratio of 1:1 via greedy or nearest neighbor matching within PS scores of 0.5. This strategy resulted in 48 matched pairs in each group. All tests were two-tailed, and *P*-values of < 0.05 were considered statistically significant.

## Results

### Clinical characteristics of patients

**Fig. 1:**
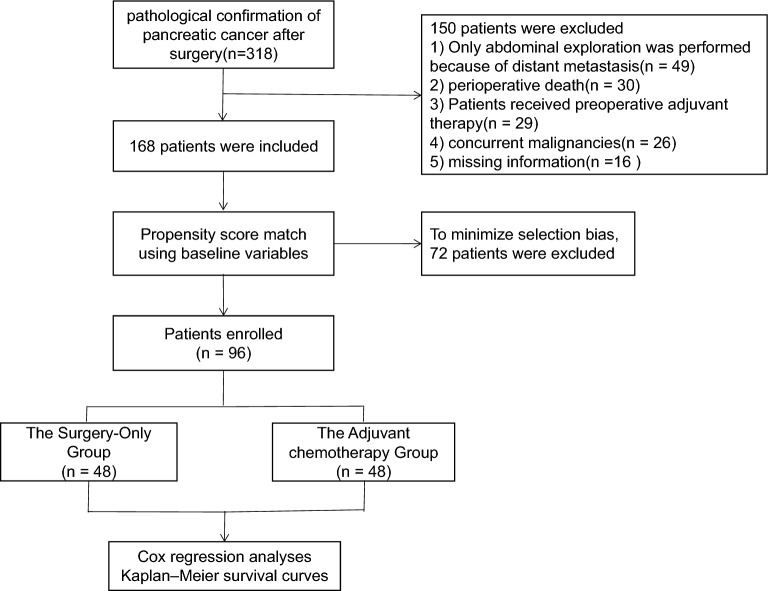
Flow chart of the patient enrolling process

A total of 168 pancreatic cancer patients were divided into two groups. Among them, 119 patients (70 males, 49 females) underwent surgery followed by adjuvant chemotherapy (AC group), while 49 patients (24 males, 25 females) underwent surgery alone (no-AC group). After PSM implementation, a total of 96 patients were included, with 48 patients in both the no-AC and AC groups. Detailed clinical characteristics of patients are presented in Table 1. The process of inclusion, exclusion, grouping, matching, and analysis of patients with pancreatic cancer in this study is demonstrated in Fig. 1.

**Table 1:** Baseline clinicopathological characteristics based on Adjuvant chemotherapy, before and after propensity score matching

Variables	Total cohort		X^2^/Z	P	Propensity score-matched cohort		X^2^/Z	P
	no-AC (n = 49)	AC (n = 119)			no-AC (n = 48)	AC (n = 48)		
Gender								
Male	24	70			24	27		
Female	25	49	1.365	0.243	24	21	0.376	0.539
Age(years)								
< = 55	13	41			12	17		
> 55	36	78	0.999	0.318	36	31	1.235	0.266
Pathological diagnosis								
Ductal adenocarcinoma	30	93			29	34		
Acinar cell carcinoma	5	7			5	2		
Other	14	19	5.073	0.079	14	12	1.836	0.399
Tumor differentiation								
Low	14	56			13	22		
Moderate	28	49			28	21		
High	7	14	4.954	0.084	7	5	3.648	0.161
Tumor size(cm)								
< = 5 cm	29	81			29	34		
> 5 cm	19	38	0.890	0.345	19	14	1.154	0.283
Microvascular invasion								
Absence	32	85			32	33		
Presence	17	34	0.615	0.433	16	15	0.068	0.827
Nerve invasion								
Absence	18	22			18	6		
Presence	31	97	6.371	0.012	30	42	4.000	0.105
Preoperative Ca19-9(u/ml)								
≤ 37.00	18	32			18	11		
> 37.00	31	87	1.609	0.205	30	37	2.421	0.120
Preoperative CEA(ng/mL)								
≤ 5.00	39	65			38	23		
> 5.00	10	54	9.176	0.002	10	25	3.117	0.101
Preoperative TBIL(umol/L)								
≤ 17.10	35	96			35	37		
> 17.10	14	23	1.727	0.189	13	11	0.222	0.637
Tumor location								
Head and neck of pancreas	12	26			11	6		
Body and tail of pancreas	37	93	0.138	0.710	37	42	1.787	0.181
AJCC 8th Stage								
I	21	18			21	14		
II	15	73			14	24		
III	13	28	9.345	0.005	13	10	4.423	0.110
Lymphatic metastasis								
Absence	37	68			36	25		
Presence	12	51	4.996	0.025	12	23	3.441	0.160
Surgical type								
Open surgery	33	78			32	28		
Laparoscopic surgery	7	25			7	13		
Da Vinci Surgical System	9	16	1.406	0.495	9	7	2.317	0.314
Revascularization								
Absence	37	104			36	42		
Presence	12	15	3.635	0.057	12	6	2.462	0.117
Operation time (min)								
≤ 180	12	17			12	7		
> 180	37	102	2.530	0.112	36	41	1.640	0.200
Amount of bleeding(mL)								
≤ 400	33	82			33	32		
> 400	16	37	0.039	0.843	15	16	0.048	0.827
Perioperative blood transfusion								
Absence	29	88			29	32		
Presence	20	31	3.579	0.058	19	16	0.405	0.525
Surgical margin								
R0	42	95			41	41		
R1/R2	7	24	0.798	0.372	7	7	0.000	1.000
Postoperative pancreatic fistula								
Absence	37	88			36	34		
Presence	12	31	0.044	0.833	12	14	0.211	0.646
Postoperative hospitalization time(days)								
≤ 14	39	83			38	33		
> 14	10	36	1.692	0.193	10	15	1.352	0.245

### Survival analysis in the AC and no-AC groups

**Fig. 2:**
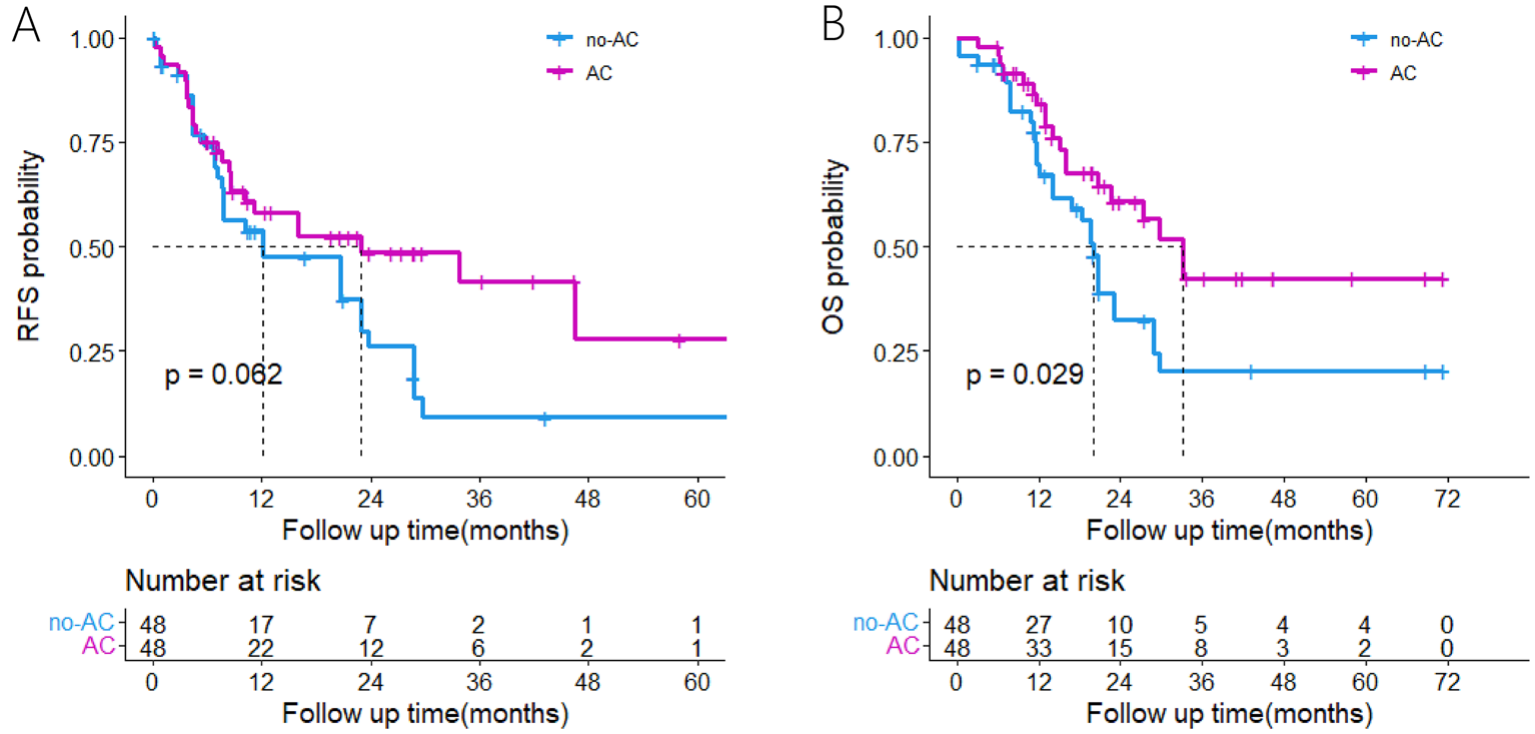
Kaplan–Meier analyses for RFS and OS based on adjuvant chemotherapy. **A**, RFS in the AC group versus the no-AC group; **B**, OS in the AC group versus the no-AC group

No significant difference was observed in RFS between the AC and no-AC groups (P = 0.062); however, OS was significantly better in the AC group than in the no-AC group (P = 0.029). Kaplan–Meier survival curves are demonstrated in Fig. 2.

### Prognostic factors for RFS and OS analyzed via Cox regression

The results of multivariate analysis revealed that lymphatic metastasis (P = 0.001), type of surgery (P = 0.015), amount of bleeding (P = 0.011), and surgical margin (P = 0.017) were independent prognostic factors for RFS. Additionally, age (P = 0.016), pathological diagnosis (P = 0.010), and lymphatic metastasis (P = 0.047) were independent prognostic factors for OS. (Variables with P-values < 0.05 in the univariate analysis were included in the multivariate analysis.) Detailed results are presented in Table 2.

**Table 2:** Univariate and multivariate analysis of RFS and OS in the cohort after PSM

Characteristics		RFS						OS					
		Univariate analyses			Multivariate analyses			Univariate analyses			Multivariate analyses		
		HR	95%CI	P value	HR	95%CI	P value	HR	95%CI	P value	HR	95%CI	P value
Gender	(Male vs female)	0.95	0.55–1.66	0.869				0.62	0.33–1.18	0.143			
Age(years)	(< = 55 vs > 55)	1.47	0.76–2.84	0.253				2.39	1.20–4.75	0.013	2.33	1.17–4.64	0.016
Pathological diagnosis	Ductal adenocarcinoma	Ref.						Ref.					
	Acinar cell carcinoma	0.64	0.21–1.90	0.420				1.07	0.40–2.80	0.890	1.25	0.46–3.40	0.654
	Other	0.92	0.49–1.73	0.810				0.32	0.14–0.72	0.006	0.33	0.14–0.77	0.010
Tumor differentiation	Low	Ref.						Ref.					
	Moderate	0.77	0.43–1.39	0.402				0.75	0.40–1.41	0.380			
	High	1.04	0.41–2.60	0.932				0.53	0.15–1.81	0.313			
Tumor size(cm)	(< = 5.00 vs > 5.00)	0.89	0.49–1.61	0.706				0.58	0.29–1.18	0.132			
Microvascular invasion	(Absence vs presence)	0.98	0.54–1.77	0.951				0.65	0.35–1.21	0.176			
Nerve invasion	(Absence vs presence)	1.14	0.57–2.28	0.719				1.08	0.50–2.34	0.852			
Preoperative Ca19-9(u/ml)	(≤ 37.00 vs > 37.00)	1.55	0.79–3.04	0.201				1.61	0.77–3.35	0.205			
Preoperative CEA(ng/mL)	(≤ 5.00 vs > 5.00)	1.34	0.77–2.33	0.294				1.24	0.70–2.20	0.465			
Preoperative TBIL(umol/L)	(≤ 17.10 vs > 17.10)	1.78	0.99–3.18	0.053				1.32	0.72–2.43	0.373			
Tumor location	(Head and neck of pancreas vs Body and tail of pancreas)	1.65	0.70–3.87	0.252				0.80	0.38–1.67	0.547			
AJCC 8th stage	I(T_1_N_0_M_0,_ T_2_N_0_M_0_)	Ref.						Ref.					
	II(T_3_N_0_M_0,_ T_1~3_N_1_M_0_)	1.21	0.63–2.32	0.549	1.20	0.63–2.30	0.566	0.87	0.44–1.70	0.690			
	III(T_4_N_0~2_M_0,_ T_1~4_N_2_M_0_)	2.11	1.01–4.40	0.046	1.83	0.87–3.86	0.110	1.36	0.66–2.78	0.394			
Lymphatic metastasis	(Absence vs presence)	2.28	1.28–4.06	0.005	2.75	1.52–4.95	0.001	1.97	1.04–3.74	0.037	1.90	1.01–3.59	0.047
Surgical type	Open surgery	Ref						Ref					
	Laparoscopic surgery	2.27	1.21–4.25	0.010	2.17	1.16–4.06	0.015	2.19	1.05–4.58	0.036	2.11	1.00–4.48	0.050
	da Vinci Surgical system	0.59	0.23–1.52	0.278	0.63	0.24–1.65	0.355	0.98	0.40–2.39	0.980	1.16	0.47–2.86	0.746
Revascularization	(Absence vs presence)	1.18	0.61–2.30	0.625				1.47	0.73–2.99	0.282			
Operation time(min)	(≤ 180 vs > 180)	1.67	0.78–3.58	0.186				1.20	0.60–2.37	0.609			
Amount of bleeding(mL)	(≤ 400 vs > 400)	2.11	1.20–3.71	0.009	2.12	1.19–3.79	0.011	1.31	0.71–2.40	0.389			
Perioperative blood transfusion	(Absence vs presence)	1.61	0.93–2.78	0.090				1.01	0.56–1.80	0.979			
Surgical margin	(R0 vs R1/R2)	2.42	1.21–4.87	0.013	2.42	1.17–5.01	0.017	1.55	0.77–3.15	0.222			
Postoperative pancreatic fistula	(Absence vs presence)	1.03	0.55–1.93	0.938				1.70	0.91–3.19	0.095			
Hospitalization time (days)	(≤ 14 vs > 14)	1.17	0.63–2.17	0.610				0.67	0.34–1.33	0.259			

### Survival analysis of patients with pancreatic cancer of different AJCC 8th edition stages

**Fig. 3:**
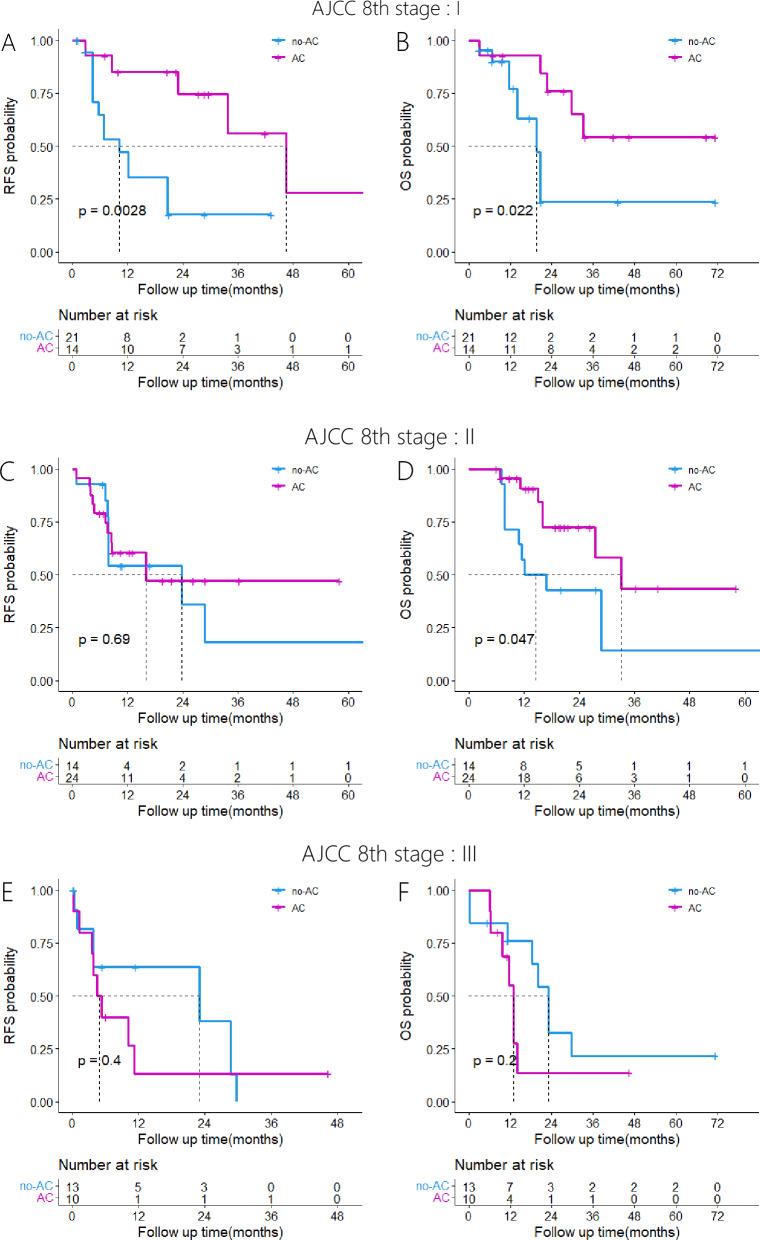
Kaplan–Meier analyses for RFS and OS based on adjuvant chemotherapy. **A**, RFS in the AC group versus the no-AC group in patients with AJCC 8th edition stage I pancreatic cancer; **B**, OS in the AC group versus the no-AC group in patients with AJCC 8th edition stage I pancreatic cancer; **C**, RFS in the AC group versus the no-AC group in patients with AJCC 8th edition stage II pancreatic cancer; **D**, OS in the AC group versus the no-AC group in patients with AJCC 8th edition stage II pancreatic cancer; **E**, RFS in the AC group versus the no-AC group in patients with AJCC 8th edition stage III pancreatic cancer; **F**, OS in the AC group versus the no-AC group in patients with AJCC 8th edition stage III pancreatic cancer

Among patients diagnosed with stage I pancreatic cancer according to the AJCC 8th edition, the AC group exhibited significantly improved RFS and OS compared to the no-AC group (P = 0.0028; P = 0.022). Among patients diagnosed with stage II pancreatic cancer, no significant difference in RFS was observed between the AC and no-AC groups (P = 0.69). However, the AC group exhibited significantly improved OS compared to the no-AC group (P = 0.047). Among patients diagnosed with stage III pancreatic cancer, no significant difference was observed in terms of RFS and OS between the AC and no-AC groups (P = 0.40; P = 0.20). Kaplan–Meier survival curves are shown in Fig. 3.

### Survival analysis of patients with lymphatic metastasis

**Fig. 4:**
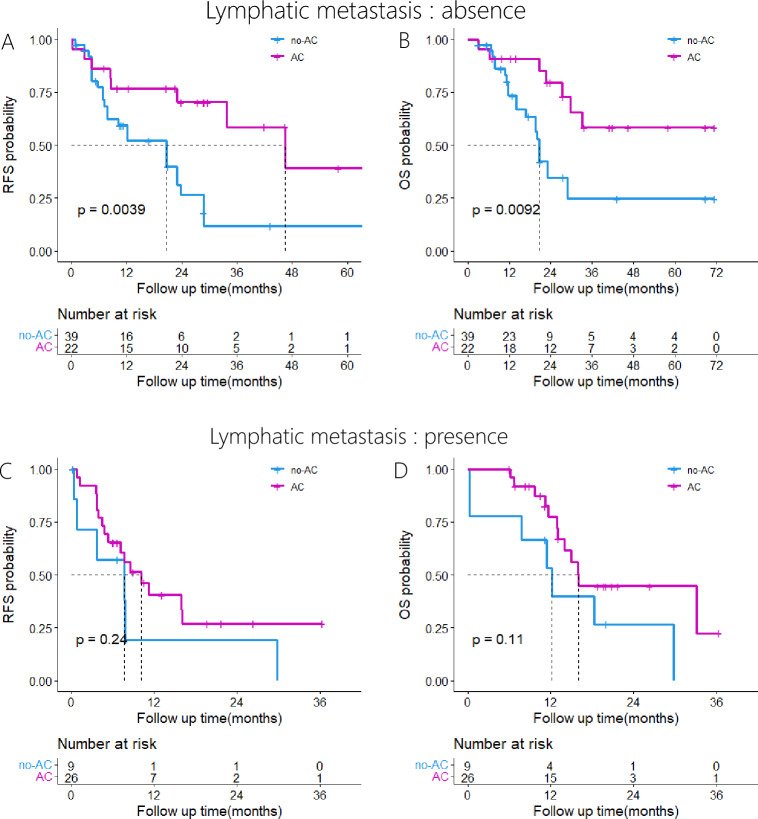
Kaplan–Meier analyses for RFS and OS based on adjuvant chemotherapy. **A**, RFS in the AC group versus the no-AC group in patients without lymph node metastasis; **B**, OS in the AC group versus the no-AC group in patients without lymph node metastasis; **C**, RFS in the AC group versus the no-AC group in patients with lymph node metastasis; **D**, OS in the AC group versus the no-AC group in patients with lymph node metastasis

Among patients without lymph node metastasis, the AC group demonstrated significantly improved RFS and OS compared to the no-AC group (P = 0.0039; P = 0.0092). No significant disparity in RFS and OS was detected between the AC and no-AC groups among patients afflicted with lymph node metastasis (P = 0.24; P = 0.11). Kaplan–Meier survival curves are shown in Fig. 4.

### Analysis of adverse events in patients who received adjuvant chemotherapy

A comprehensive evaluation of adverse effects was conducted on the cohort of 119 patients in this study. Among the cohort of 119 patients, 39 were administered GP, 29 received mFOLFIRINOX, and 51 underwent S-1 monotherapy. In the GP group, the predominant adverse reactions included nausea (48.7%), rash (46.2%), and diarrhea (43.6%). Within the mFOLFIRINOX group, the prevailing adverse events comprised nausea (51.7%), elevated TBIL levels (48.3%), and diarrhea (44.8%). As for the S-1 group, the primary adverse reactions observed were diarrhea (49.0%), nausea (47.1%), and rash (43.1%). All patients experiencing grade 1–2 adverse reactions exhibited improvement through careful observation or symptomatic treatment. The majority of patients experiencing grade 3–4 adverse reactions exhibited improvement following drug dose reduction, withdrawal, and symptomatic treatment. Within the mFOLFIRINOX group, one patient experienced severe thrombocytopenia, which resolved after one week of drug withdrawal and platelet infusion. There were no notable disparities in the occurrence of grade 3–4 adverse reactions among the three chemotherapy groups (P > 0.05 for all), with the frequency of such reactions being less than 25.6%. No deaths occurred as a result of adverse reactions throughout the entire study cohort. Detailed information is presented in Table 3.

**Table 3:** Postoperative adjuvant chemotherapy related adverse reactions

Adverse Event	CTCAE v5.0 grade 1–2					CTCAE v5.0 grade 3–4				
	GP (n = 39)	mFOLFIRINOX (n = 29)	S−1 (n = 51)	Z	P	GP (n = 39)	mFOLFIRINOX (n = 29)	S−1 (n = 51)	Z	P
Nausea	19 48.7%	15 51.7%	24 47.1%	0.161	0.923	10 25.6%	5 17.2%	8 15.7%	1.512	0.470
Diarrhea	17 43.6%	13 44.8%	25 49.0%	0.292	0.864	5 12.8%	3 10.3%	5 9.8%	0.220	0.896
Rash	18 46.2%	8 27.6%	22 43.1%	2.674	0.263	2 5.1%	3 10.3%	3 5.9%	0.822	0.663
Hand-foot syndrome	12 30.8%	8 27.6%	22 43.1%	2.478	0.290	5 12.8%	4 13.8%	4 7.8%	0.887	0.642
Fatigue	13 33.3%	11 37.9%	21 41.2%	0.578	0.749	5 12.8%	5 17.2%	4 7.8%	1.635	0.441
Leucopenia	13 33.3%	13 44.8%	20 39.2%	0.938	0.625	4 10.3%	4 13.8%	7 13.7%	0.291	0.865
Anemia	9 23.1%	10 34.5%	9 17.6%	2.919	0.232	7 17.9%	4 13.8%	6 11.8%	0.698	0.705
Thrombocytopenia	14 35.9%	9 31.0%	11 21.6%	2.337	0.311	2 5.1%	1 3.4%	3 5.9%	0.230	0.892
ALT elevation	7 17.9%	12 41.4%	7 13.7%	8.797	0.012	4 10.3%	4 13.8%	5 9.8%	0.329	0.848
AST elevation	6 15.4%	12 41.4%	8 15.7%	8.567	0.014	3 7.7%	0 0.0%	5 9.8%	2.921	0.232
TBIL elevation	7 17.9%	14 48.3%	14 27.5%	7.534	0.023	4 10.3%	0 0.0%	2 3.9%	3.888	0.143
Proteinuria	10 25.6%	12 41.4%	13 25.5%	2.645	0.266	0 0.0%	2 6.9%	2 3.9%	2.522	0.283
Hematuresis	5 12.8%	11 37.9%	9 17.6%	6.927	0.031	3 7.7%	0 0.0%	1 2.0%	3.569	0.168
Creatinine elevation	7 17.9%	12 41.4%	10 19.6%	6.052	0.049	4 10.3%	1 3.4%	2 3.9%	2.012	0.366

## Discussion

The viability and precision of the American Joint Committee on Cancer (AJCC) 8th edition staging system for pancreatic cancer have been validated through several notable large-scale studies [7, 8]. Moreover, adjuvant chemotherapy has been demonstrated to significantly prolong the survival time of pancreatic cancer patients. The CONKO-001 study, a captivating multicenter, randomized controlled phase III clinical trial encompassing 368 patients [1], demonstrated that patients who received surgery combined with gemcitabine-based adjuvant chemotherapy experienced a remarkable extension in disease-free survival (DFS) and OS compared to those who underwent surgery alone. Notably, the median DFS reached an impressive 13.4 months compared to 6.7 months (P < 0.001). Subsequent studies [2, 3, 9] proposed alternative approaches apart from using gemcitabine as a standalone treatment. However, the benefits of adjuvant chemotherapy over surgery alone for pancreatic cancer patients stratified by AJCC staging remain uncertain. Further research is still needed to explore the relationship between the efficacy of adjuvant chemotherapy and AJCC staging in pancreatic cancer. Gervaso et al. [10] emphasized in a comprehensive review that the decision regarding adjuvant therapy in stage I pancreatic cancer patients remains a challenge, necessitating further data to draw definitive conclusions.

The findings of this study may potentially address this crucial issue. This study unveiled that in stage I pancreatic cancer patients, the AC group demonstrated significantly enhanced RFS and OS in comparison to the no-AC group (P = 0.0028; P = 0.022). Similarly, Turner et al. [11] demonstrated that patients diagnosed with stage IA pancreatic cancer who received adjuvant chemotherapy experienced a significantly prolonged median OS of 105.7 months, surpassing those who underwent surgery alone (72.0 months) (P < 0.01). Furthermore, Guenther et al. [12] observed analogous findings in a cohort of 124 patients with stage I pancreatic cancer. Interestingly, Izumo et al. [13] reported seemingly disparate findings. They stratified stage IA pancreatic cancer patients into low-risk and high-risk groups based on clinical and pathological factors, revealing that adjuvant chemotherapy exhibited greater efficacy in the high-risk group. Moreover, it is imperative to acknowledge that the duration of this study, spanning from 2018 to 2021, was comparatively shorter than that of other studies, effectively mitigating the heterogeneity of treatment regimens. These results illustrate that the benefit of postoperative adjuvant chemotherapy varies according to AJCC stage, and the standard chemotherapy regimen should be questioned whether it is appropriate for each patients. For patients with stage I and II pancreatic cancer, current standard adjuvant chemotherapy regimens are relatively effective. However, the survival of patients with stage III pancreatic cancer from current postoperative chemotherapy is relatively modest, suggesting that more aggressive chemotherapy regimens may be needed or that clinical trials will be conducted to identify more effective regimens.

Considering the inherent biases associated with retrospective, single-center data, we endeavored to mitigate this limitation by employing a PSM approach. PSM is a statistical methodology employed in observational studies, alleviating the impact of selection bias and confounding variables, thereby rendering the comparison between the experimental and control groups more robust [14, 15]. Nevertheless, it is crucial to acknowledge several limitations of this study. Firstly, we did not explore the impact of retreatment after cancer recurrence on OS. Secondly, the prognostic significance of histological differentiation in pancreatic cancer has been widely recognized; however, we did not stratify and analyze survival based on this factor. Lastly, the sample size was limited, and all participants were from southern China, potentially compromising the generalizability of the study population. The conclusions of this study need to be verified by external data from different populations and regions, and only in this way can the conclusions of this study be convincing enough.

In conclusion, our study findings indicate the beneficial effects of adjuvant chemotherapy on both RFS and OS in stage I pancreatic cancer patients. For stage II pancreatic cancer, adjuvant chemotherapy improves OS but does not affect RFS. However, in stage III pancreatic cancer, adjuvant chemotherapy fails to improve both RFS and OS. Therefore, further clinical research is warranted to establish effective adjuvant chemotherapy regimens specifically tailored for stage III pancreatic cancer patients.

## Data Availability

The data used to support the findings of this study are included in the article.
